# Effect of high-flow nasal therapy during early pulmonary rehabilitation in patients with severe AECOPD: a randomized controlled study

**DOI:** 10.1186/s12931-020-1328-z

**Published:** 2020-04-15

**Authors:** Lan-Fang Tung, Sheng-Yeh Shen, Hui-Hsuan Shih, Yen-Ting Chen, Chia-te Yen, Shu-Chuan Ho

**Affiliations:** 1 Division of Pulmonary Medicine, Department of Internal Medicine, MacKay Memorial Hospital, Taipei City, Taiwan; 2 School of Respiratory Therapy, College of Medicine, Taipei Medical University, 250 Wuxing Street, Taipei, 11031 Taiwan; 3 Division of Pulmonary Medicine, Department of Internal Medicine, Shuang Ho Hospital, Taipei Medical University, New Taipei City, Taiwan

**Keywords:** Chronic obstructive pulmonary disease, Pulmonary rehabilitation, CRP, Dyspnea sensation, High-flow nasal therapy, Six-minute walking distance

## Abstract

**Background:**

Chronic obstructive pulmonary disease (COPD) is airway inflammation characterized and low daily physical activity. Most pulmonary rehabilitation (PR) programs are often provided to stable patients, but fewer training programs are specific for hospitalized patients with acute exacerbation (AE). Patients with AECOPD experience increased dyspnea sensations and systemic inflammation during exercise training. High-flow nasal therapy (HFNT) reduces the minute volume, lowers the respiratory rate, and decreases the work of breathing. However, it is not clear whether HFNT is efficient during exercise training. In this study, we investigated the effects of HFNT during exercise training in an early PR program among hospitalized patients with severe AECOPD.

**Methods:**

We enrolled COPD patients hospitalized due to AE. They were randomized into two groups according to their status into HFNT PR and non-HFNT PR groups. This study collected basic data, and also assessed a pulmonary function test, 6-min walking test, blood inflammatory biomarkers, and arterial gas analysis at the baseline, and at 4 and 12 weeks of the intervention. Data were analyzed using SPSS statistical software.

**Result:**

We recruited 44 AECOPD patients who completed the 12-week PR program. The HFNT PR program produced significant improvements in exercise tolerance as assessed by the 6-min walking distance (6MWD), reduced dyspnea sensations in the modified Medical Research Council (mMRC), and decreased systemic inflammation as evidenced by the a lower C-reactive protein (CRP) level. A reduction in the length of hospitalization was achieved with PR in the 1-year follow-up in the two groups. The HFNT PR group showed better trends of reduced air trapping in the delta inspiration capacity (IC) and an increased quality of life according to the COPD assessment test (CAT) than did the non-HFNT PR group.

**Conclusions:**

HFNT during exercise training in early PR increases exercise tolerance and reduces systemic inflammation in hospitalized patients with severe AECOPD.

## Introduction

Chronic obstruction pulmonary disease (COPD) patients have lower daily physical activity [1], and are also characterized by low-grade systemic inflammation caused by circulating inflammatory mediators. A past study found that intracellular oxidative stress was increased in patients with severe COPD [2]. Acute exacerbation (AE) of COPD (AECOPD) is defined as episodes of acute worsening of respiratory symptoms (such as dyspnea, coughing, and sputum production) that require additional therapy [3, 4]. Treatments for AECOPD aim to minimize the negative impacts of the current exacerbation and prevent subsequent events, such as relapse or readmission to the hospital [5]. AECOPD requiring hospitalization is associated with poor outcomes, including accelerated declines in muscle strength [6] and lung function [7], a reduced health status and quality of life (QOL) [8], accelerated disease progression [9], a significant risk of recurrent exacerbations, and an increased risk of mortality [10, 11]. It was reported in the United Kingdom that 43% of patients hospitalized with AECOPD were readmitted and 12% died within 90 days. They account for more than 70% of all COPD-related costs [12], and AECOPD is responsible for patients’ clinical deterioration. In this way, treatment goals for patients with AECOPD are to minimize the negative impacts of these events and prevent their recurrence [13]. The management of severe AECOPD is divided into pharmacological (inhaled bronchodilators, steroids, and antibiotics) and non-pharmacological treatments (oxygen therapy, high-flow nasal therapy (HFNT), non-invasive mechanical ventilation (NIMV), and pulmonary rehabilitation (PR)) [5].

PR is a comprehensive non-pharmacological treatment which has the best chance of improving COPD symptoms [14]. According to an American Thoracic Society (ATS)/European Respiratory Society statement, PR is a cornerstone intervention for managing patients with stable COPD [13]. PR is a comprehensive intervention that includes exercise training, education, and behavioral changes [15]. PR benefits COPD patients at different functional stages [16] and with different phenotypes [17]. PR programs are most often provided to stable patients or after discharge [18], and fewer training programs specific to AE in unstable periods during hospitalization have been developed. Most studies that assessed the efficiency of PR during AECOPD have shown controversial results in hospitalized patients [12], who present more-severe exacerbation and/or more-severe underlying disease than stable COPD patients in an outpatient setting. The use of systemic steroids [19] and NIMV [20] for in-hospital AECOPD is supported by strong evidence of their efficacy. Some AECOPD patients are unable to reach the required training intensity to improve clinical or physiological effects. HFNT can deliver up to 60 L/min of reheated, humidified air via a nasal cannula. It can increase alveolar ventilation, improve arterial blood gas (ABG) data, and reduce the effort of breathing [21–23]. HFNT in stable hypercapnia patients leads to a flow-dependent reduction in the hypercapnia level [21]. But, HFNT needs to be assessed in further prospective studies, especially when combined with exercise training and PR programs.

Therefore, this study aimed to investigate the effects of the combination of HFNT with exercise training in an early PR program in patients with AECOPD and assess its effects on hospitalization times, AECOPD patient symptoms, exercise tolerance, QOL, and functionality.

## Materials and methods

### Study subjects

**Fig. 1:**
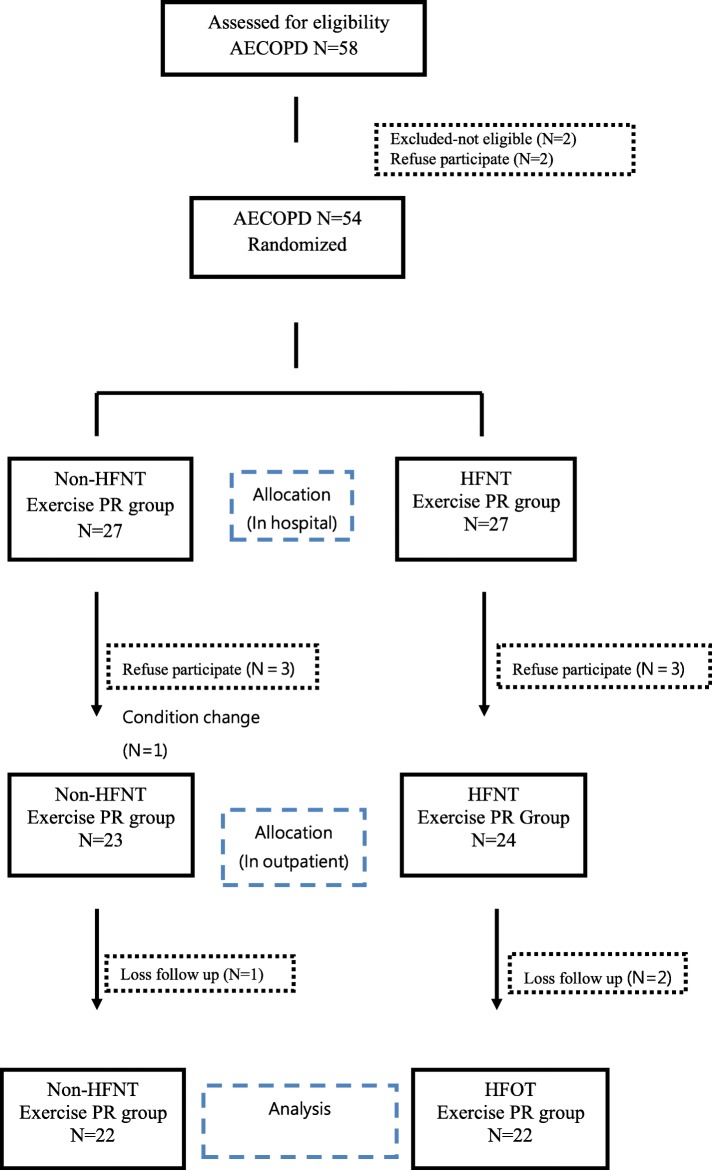
Flow diagram of participants through the study

We enrolled 54 patients with COPD who were hospitalized due to AE in Mackay Memorial Hospital (Taipei City, Taiwan) between April 2017 and April 2019; ten of them did not finish the course (Fig. 1). Patients who had received a diagnosis of COPD exhibited a post-bronchodilator forced expiratory volume in the first second (FEV1)/forced vital capacity (FVC) ratio of ≤70%. All COPD patients were in category C or D according to the combined COPD assessment using new GOLD guidelines [24]. All participants were aged 40~90 years and were ambulatory by 48 h after admission for AECOPD at the time of inclusion. Subjects are required to do every day during hospitalization, and continued twice a week until 3 months after discharge. Study subjects were then followed for 12 months. Subjects with known unstable vital signs (including a body temperature of > 38.5 °C, a respiration rate (RR) of > 40 beats/min or < 10 beats/min, and heart rate (HR) of > 150 beats/min or < 40 beats/min, and a mean blood pressure (BP) of < 70 mmHg), a malignant tumor, angina, myocardial infarction, severe hypoxemia, an unstable psychological status, hemoptysis, and pneumothorax were excluded. There was no significant adverse event during the rehabilitation program, except one participate who withdrew the program because of progressive dyspnea during lower limb exercise. All study participants provided written informed consent (17MMHIS012).

### Study design

This was a prospective, cross-section, randomized controlled study in which subjects were randomized to an HFNT PR group and a non-HFNT PR group. The HFNT PR group received HFNT during exercise training in addition to the usual care and PR program. The non-HFNT PR group only received usual care and a PR program. All patients attended a 12-week, twice-a-week, in-hospital, multidisciplinary PR program, consisting of four components: (a) health education; (b) breathing exercises; (c) bronchial hygiene and lung expansion therapy; and (d) exercise training.

#### Health education

All patients received information from a respiratory therapist about disease awareness, the proper use of medications, pathophysiology, muscle relaxation and energy conservation methods, bronchial hygiene methods, how to cope with acute dyspnea, and nutritional guidance.

#### Breathing exercises

All patients were taught to use a pursed-lip/diaphragmatic breathing exercise during limb muscle training, pacing waking, and bronchial hygiene.

#### Bronchial hygiene

Postural drainage, percussion, vibration, and effective coughing techniques to improve airway clearance were carried out regularly daily at home and weekly at hospital visits in case of sputum impaction. At the weekly follow-up clinical visit, if needed, patients were provided with lung expansion therapy with intermittent positive pressure breathing or a negative-pressure ventilator.

#### Exercise training

Different types of physical exercises were used, for example, limb strength training, and ergometer bicycle training. These were constant-work-rate exercises, including warm-up, rapid-pace walking, and cool-down. At the twice-weekly follow-up clinic visits, patients underwent upper- and lower-limb bicycle ergometer training for 20 min, and perceived symptoms of breathlessness and muscle fatigue.

#### HFNT

The AIRVO 2 system (Fisher & Paykel, Auckland, New Zealand) was used to provide HFNT. This system generates humidified air. The flow was set to 50 L/min and provide an extended range of oxygen concentrations when needed during the exercise. The patient was instructed to breathe through the nose.

### Data collection

Each participant was interviewed by a well-trained respiratory therapist to collect demographic, lifestyle, and disease-related data (smoking habits, number of exacerbations in the past year, medications used in the stable and exacerbation periods of the disease, and comorbidities) which were collected within 48 h of AECOPD onset, after the 4-week PR follow-up clinical visit, and at the end of the 12-week PR training period. After the end of the 12-week study, observational AE data were collected at a 1-year follow-up assessment. The body-mass index (BMI) was calculated as the weight divided by the height squared (kg/m^2^). The modified Medical Research Council (mMRC) scale, graded from 0 to 4, is a simple, valid and widely used instrument to characterize the impact of dyspnea on daily activities of patients with COPD [25, 26]. Variations of 0.6 units [27] were indicated to be a the minimal clinically important difference (MCID) for patients with AECOPD after pharmacological treatment. The impact of the disease was measured with the previously validated Chinese version of the COPD Assessment Test (CAT), and an MCID of 2 points for patients with AECOPD receiving pharmacological treatment was previously established [28].

Pulmonary function parameters were assessed using a Vitalograph Spirotac V™ (Vitalograph, England) after a 10-min rest; the post-bronchodilator FEV1 and FVC were measured, and the FEV1/FVC ratio was calculated. The exercise capacity was assessed with a 6-min walking test (6MWT), according to ATS guidelines [29]. All subjects were instructed to walk as far as possible but were allowed to stop and rest during the test. Oxygen saturation and the pulse rate were recorded using a continuous finger-adapted pulse oximeter during the 6MWT. Additionally, at the beginning and the end of the 6MWT, the difference in exertion was assessed with the modified Borg’s scale (10-point scale), for rating perceived exertion by measuring breathlessness. All patients were familiar with the 6MWT before the study.

Blood samples were collected and analyses included arterial blood gas (ABG) (pH, partial pressure of oxygen (PaO_2_), PaCO_2_ and HCO_3_^−^), white blood cells (WBCs), and C-reactive protein (CRP) within 48 h after admission, at the 4-week follow-up clinical visit, and at the end of the 12-week PR training period.

### Statistical analysis

Results were statistically analyzed with SPSS for Windows 19.0 (SPSS, Chicago, IL, USA) and the program Graph-Pad Prism 5 (GraphPad Software, La Jolla, CA, USA). Descriptive analyses were performed to evaluate demographic and clinical characteristics of patients. Descriptive data are expressed as the mean ± standard deviation (SD). Continuous variables were compared using a repeated-measures test, an analysis of variance (ANOVA) was used to compare data within groups, and an unpaired *t*-test was used to compare two groups with respect to delta mMRC, BMI, obstruction, dyspnea, exercise capacity (BODE health index: body-mass index, degree of airway obstruction and dyspnea, and exercise capacity), and CAT. Categorical variables were compared between two groups using a Chi-squared test. Statistical significance was defined as *p* < 0.05.

## Results

### Patient characteristics

In total, 44 patients finished the PR course. Twenty-two patients (15 males and seven females, mean age 73.1 ± 6.4 years; FEV1: 36.5% ± 10.3% predicted) in the non-HFNT PR group, and 22 patients (17 males and five females, mean age 72.3 ± 7.7 years; FEV1: 36.6% ± 8.0% predicted) in the HFNT PR group were enrolled. Baseline characteristics of both groups are shown in Table 1. With the exception of the smoking status, mMRC, CAT, and BODE there were no significant differences between the two groups. Patients in both the non-HFNT PR and HFNT PR groups had poor exercise tolerance (6MWD, 128.5 ± 85.6 vs. 177.0 ± 89.2 m), more-severe dyspnea sensation (mMRC, 3.5 ± 0.5 vs. 3.1 ± 0.4), and a worse health status (CAT, 30.9 ± 2.1 vs. 28.9 ± 2.8). Other features were increased respiratory infection and inflammation (WBCs, 14.8 ± 5.2 vs. 14.7 ± 4.4 10^3^/μl; CRP, 8.4 ± 6.8 vs. 5.1 ± 5.6 mg/dl). pH 7.36 was in the normal range, but PaCO_2_ was above normal on the acidosis side of normal. The number of hospital admissions for AE in the previous year exceeded two times; most patients had comorbidities, especially cardiovascular diseases. The mean AE hospital length of stay was 8.46 ± 1.61 days.

**Table 1:** Characteristics of the patients with acute exacerbation of chronic obstructive pulmonary disease (AECOPD) patients in the high-flow nasal therapy pulmonary rehabilitation (HFNT PR) and non-HFNT PR groups

Characteristic	Non-HFNT PR (*N* = 22)	HFNT PR (*N* = 22)	*p* value
Age, years	73.1 ± 6.4	72.3 ± 7.7	0.703
Gender, M (%)	15 (77.3)	17 (77.3)	0.728^#^
**Smoking status**			**< 0.001**^**#**^
Non-smoker, *n* (%)	1 (4)	3 (13)	
Current smoker, *n* (%)	5 (22)	0 (0)	
Ex-smoker, *n* (%)	16 (72)	19 (86)	
Body-mass index, kg/m^2^	19.8 ± 3.1	20.3 ± 3.4	0.614
Pulmonary function test (PFT)			
FVC, % predicted normal	80.0 ± 27.8	74.3 ± 16.2	0.412
FEV_1_, % predicted normal	36.5 ± 10.3	36.6 ± 8.0	0.911
FEV_1_/FVC, %	39.6 ± 12.8	40.2 ± 11.0	0.851
Delta IC, L	−0.22 ± 0.18	−0.18 ± 0.17	0.513
6MWT			
6MWD, m	128.5 ± 85.6	177.0 ± 89.2	0.0524
SpO_2_ pre/post 6MWT, %	94.4/87.3	93.8/88.9	0.484/0.241
HR pre/post 6MWT, %	98.4/125.6	96.4/ 125.6	0.572/0.979
**mMRC, score**	**3.5 ± 0.5**	**3.1 ± 0.4**	**0.004**
**CAT score**	**30.9 ± 2.1**	**28.9 ± 2.8**	**0.009**
**BODE score**	**8.1 ± 1.3**	**7.1 ± 1.1**	**0.010**
Laboratory			
WBCs, 10^3^/μl	14.8 ± 5.2	14.7 ± 4.4	0.951
CRP, mg/dl	8.4 ± 6.8	5.1 ± 5.6	0.086
pH	7.36 ± 0.08	7.36 ± 0.08	0.973
PaCO_2_, mmHg	51.5 ± 24.1	52.6 ± 20.3	0.8691
PaO_2_, mmHg	63.2 ± 15.3	66.1 ± 14.7	0.513
HCO_3_^−^	24.9 ± 4.2	26.3 ± 6.0	0.386
AE hospitalization in the previous PR 1 year, time	2.6 ± 0.8	2.3 ± 0.7	0.167
Comorbidity			
CVD, *n* (%)	15 (68)	16 (72)	0.741^#^
GERD, *n* (%)	6 (27)	9 (40)	0.340^#^
Osteoporosis, *n* (%)	7 (31)	5 (22)	0.498^#^
Diabetes, *n* (%)	6 (27)	5 (22)	0.728^#^
Chronic renal failure, *n* (%)	2 (9)	3 (13)	0.635^#^
Anxiety, *n* (%)	4 (18)	3 (13)	0.680^#^
Length of hospitalization, days	8.6 ± 1.3	8.3 ± 1.9	0.459

Note: Data are presented as the mean ± SD; ^#^ Analyzed by a Chi-squared test

Abbreviations: *GOLD* Global Initiative for Chronic Obstructive Pulmonary Lung Disease; Group D: acute exacerbation (AE) ≥ 2; modified Medical Research Council (mMRC) ≥ 2, COPD assessment test (CAT) ≥ 10. FEV_1_, forced expiratory volume in the first second; *FVC* forced vital capacity; 6MWD, 6-min walking distance; *mMRC* modified Medical Research Council; *BODE* index body-mass index, degree of airflow obstruction and dyspnea, and exercise capacity; *WBCs*, white blood cells; *CRP*, C-reactive protein; *PaCO*_*2*_ partial pressure of carbon dioxide; *PaO*_*2*_ partial pressure of oxygen; *GERD* gastroesophageal reflux disease; *CVD* cardiovascular disease

### Clinical outcomes of pulmonary function, exercise tolerance, and laboratory data

In within-group comparisons of pulmonary function, FVC had significantly increased and delta IC had significantly decreased after 12 weeks in the non-HFNT PR group, and FEV1 had significantly increased after 12 weeks and delta IC had significantly decreased after 4 and 12 weeks in the HFNT PR group. By the end of the study, there was no significant difference in the pulmonary function test between the two groups (Table 2).

**Table 2:** Between and within groups differences on the pulmonary function test, 6-min walking test (6MWT), and laboratory data at the baseline, at 4 weeks, and after 12 weeks (*N* = 44)

	Non-HFNT PR			HFNT PR			Between-group test	
	Baseline (*n* = 22)	4 weeks (*n* = 22)	12 weeks (*n* = 22)	Baseline (*n* = 22)	4 weeks (*n* = 22)	12 week (*n* = 22)	4 weeks	12 weeks
							*p* value	
Pulmonary function test								
FVC, L	2.29 ± 0.76	2.38 ± 0.73^**a^	2.40 ± 0.74^**a^	2.26 ± 0.68	2.38 ± 0.69	2.42 ± 0.69	0.990	0.932
FEV_1,_ L	0.83 ± 0.24	0.84 ± 0.23	37.5 ± 39.6	0.86 ± 0.26	0.88 ± 0.26	0.92 ± 0.26^**a,b^	0.550	0.324
FEV_1_/FVC, %	39.6 ± 12.8	39.6 ± 12.5	39.6 ± 12.5	40.2 ± 11.0	40.3 ± 11.2	40.4 ± 11.6	0.840	0.833
Delta IC, L	−0.22 ± 0.18	−0.18 ± 0.16	−0.16 ± 0.15*^a^	−0.18 ± 0.17	−0.13 ± 0.12^*a^	−0.09 ± 0.13**^a^	0.146	0.099
6MWT								
6MWD, m	128.5 ± 85.6	191.4 ± 89.3	245.5 ± 103.1^**a,b^	178.9 ± 81.6	260.1 ± 87.7^**a^	304.6 ± 84.4^**a,b^	**0.014**^**c**^	**0.044**^**c**^
SpO_2_, % pre 6MWT	94.4 ± 3.4	95.0 ± 3.1	95.5 ± 2.6^*a^	93.8 ± 2.5	95.1 ± 2.4^*a^	95.6 ± 2.1^**a^	0.957	0.802
SpO_2_, % post 6MWT	87.4 ± 4.2	88.9 ± 4.9^*a^	90.5 ± 2.8^**a,b^	88.9 ± 4.4	90.7 ± 3.1^**a^	91.8 ± 2.4^**a^	0.136	0.103
HR, pre 6MWT	98.4 ± 10.7	92.1 ± 11.3^**a^	91.7 ± 10.8^**a^	96.5 ± 11.5	92.6 ± 10.5^**a^	91.3 ± 10.4^**a^	0.880	0.899
HR, post 6MWT	125.6 ± 8.1	124.4 ± 7.8	125.1 ± 7.4	125.6 ± 14.0	122.7 ± 6.4	122.0 ± 6.6	0.426	0.141
Laboratory data								
WBCs, 10^3^/μl	14.80 ± 5.22	8.73 ± 2.01**^a^	7.92 ± 1.56**^a^	14.71 ± 4.44	8.18 ± 2.65**^a^	7.06 ± 1.51**^a^	0.439	0.072
CRP, mg/dl	8.41 ± 6.77	1.73 ± 1.73**^a^	0.30 ± 0.39**^a^	5.11 ± 5.60	1.07 ± 1.57**^a^	0.07 ± 0.12**^a^	0.192	**0.020**^**c**^
pH	7.36 ± 0.08	7.40 ± 0.04^**a^	7.42 ± 0.05**^a^	7.36 ± 0.08	7.41 ± 0.05*^a^	7.42 ± 0.04**^a^	0.479	0.949
PaCO_2_, mmHg	51.5 ± 24.1	45.1 ± 15.5^*a^	45.1 ± 13.5^*a^	52.6 ± 20.3	40.1 ± 5.5*^a^	46.0 ± 17.0**^a^	0.752	0.755
PaO_2_, mmHg	63.2 ± 15.3	82.9 ± 21.0**^a^	81.9 ± 18.0**^a^	66.1 ± 14.7	82.7 ± 16.8**^a^	83.1 ± 17.3**^a^	0.976	0.825
HCO_3_^−^	24.88 ± 4.24	25.04 ± 5.82*^a^	27.35 ± 4.51*^a,b^	26.26 ± 5.98	27.15 ± 4.04	28.75 ± 4.15^*a^	0.169	0.290

Values are the mean ± SD; Delta inspiratory capacity (IC), post-6MWT IC - pre-6MWT IC

^a^ Baseline vs. 4 weeks and 12 weeks; ^b^ 4 weeks vs. 12 weeks; * *p* < 0.05, ** *p* < 0.01; ^c^ delta, 12 weeks - baseline and 4 weeks - baseline

Abbreviations: *FEV1* forced expiratory volume in the first second; *FVC* forced vital capacity; 6MWD, 6-min walking distance; *SpO*_*2*_ oxyhemoglobin saturation by pulse oximetry; *HR* heart rate; *IC* inspiratory capacity; *WBCs* white blood cells; *CRP* C-reactive protein; *PaCO*_*2*_ partial pressure of carbon dioxide; *PaO*_*2*_ partial pressure of oxygen; *HCO*_*3*_^*−*^ hydrogen carbonate bicarbonate ion

For within-group comparisons, the two groups had significantly increased exercise tolerance in the 6MWD, SpO_2_, and HR after 4 and 12 weeks of PR. However, no significant changes were found in after exercise HR in the two groups. In between-group comparisons, the 6MWD had significantly increased after 4 and 12 weeks of the HFNT PR program (Table 2). However, no significant changes were found in the SpO_2_ or HR between in two groups. There was a greater increase in exercise tolerance with HFNT PR than with non-HFNT PR among AECOPD patients.

There were significantly improved clinical conditions in terms of WBCs, CRP, pH, PaCO_2_, and PaO_2_ after 4 and 12 weeks of the intervention in both the non-HFNT PR and HFNT PR groups. The non-HFNT PR group had significantly increased HCO_3_^−^ levels after 4 and 12 weeks, while that of the HFNT PR group had only increased at 12 weeks. In between-group comparisons, laboratory data did not significantly differ in the two groups at 4 and 12 weeks of the two PR programs, except for CRP which had significantly decreased after 12 weeks in the HFNT PR group (Table 2). AECOPD patients who underwent HFNT PR had a greater decrease in the inflammation level compared to AECOPD patients in the non-HFNT PR group.

### Dyspnea sensation

**Fig. 2:**
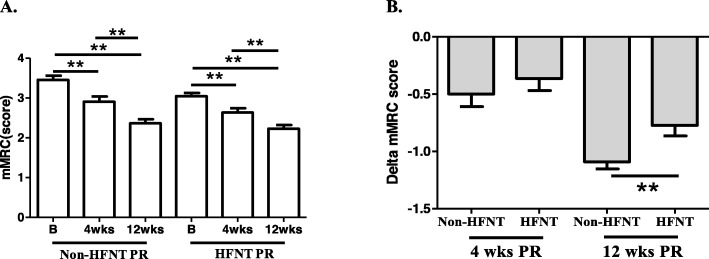
Effects of non-high-flow nasal therapy (HFNT) pulmonary rehabilitation (PR) vs. HFNT PR on the modified Medical Research Council (mMRC) scale. **a**. Individual changes in mMRC in both groups at the baseline, and after 4 and 12 weeks of the intervention. **b**. Comparisons of delta-mMRC (changes from the baseline after 4 and 12 weeks; mean ± SD) between the two groups

In within-group comparisons, the non-HFNT PR and HFNT PR groups had significantly decreased dyspnea sensations according to the mMRC after 4 and 12 weeks of the intervention (Fig. 2a). Delta mMRC had not significantly increased at 4 weeks (− 0.546 ± 0.510, − 0.409 ± 0.503) of the PR intervention in the between-group comparisons, and only delta mMRC had significantly deceased at 12 weeks (− 1.091 ± 0.294, − 0.818 ± 0.395) (Fig. 2b).

### QOL

**Fig. 3:**
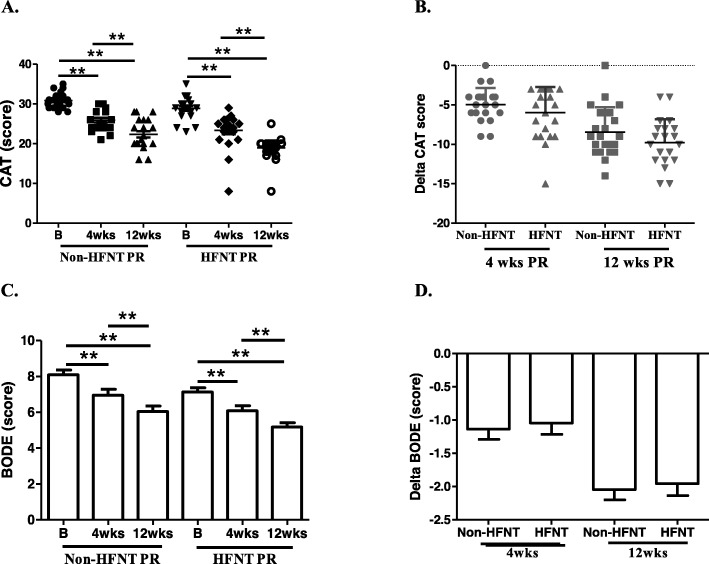
Effects of non-high-flow nasal therapy (HFNT) pulmonary rehabilitation (PR) vs. HFNT PR on chronic obstruction pulmonary disease assessment test (CAT) and the BODE index (body-mass index, degree of airway obstruction and dyspnea, and exercise capacity). **a** and **c**. Individual changes in CAT and BODE in both groups at the baseline, and after 4 and 12 weeks of the intervention. **b** and **d**. Comparisons of delta-CAT and delta-BODE (changes from the baseline after 4 and 12 weeks; mean ± SD) between the two groups

In within-group comparisons, the non-HFNT PR and HFNT PR groups had significantly improved health statuses according to CAT and BODE scores after 4 and 12 weeks of PR (Fig. 3a, c). The delta CAT (4 weeks: − 4.955 ± 2.104, − 5.545 ± 2.988; 12 weeks: − 8.500 ± 3.203, − 9.955 ± 2.984) and BODE (4 weeks: − 1.136 ± 0.710, − 1.045 ± 0.785; 12 weeks: − 2.045 ± 0.722, − 1.955 ± 0.844) scores exhibited no significant differences in between-group comparisons at 4 or 12 weeks of PR (Fig. 3b, d).

### Hospital admissions for AE

**Fig. 4:**
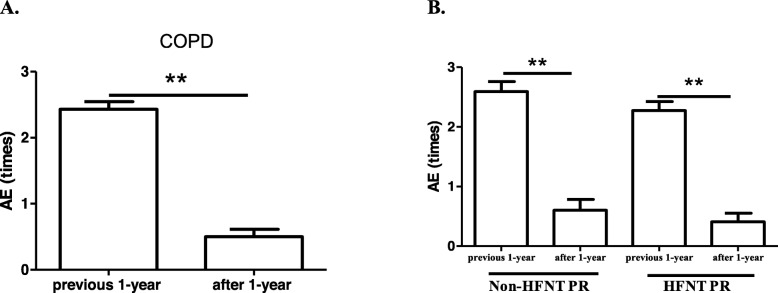
Effects of non-high-flow nasal therapy (HFNT) pulmonary rehabilitation (PR) vs. HFNT PR on hospital admissions during acute exacerbation (AE). **a**. Compared to the previous 1 year before and subsequent 1 year after the PR program in all patients. **b**. Individual changes of AE in both groups the previous year before and subsequent year after PR

Hospital admissions for AE were compared in the previous year and at 1 year after the PR program (2.43 ± 0.76 vs. 0.50 ± 0.74 times/year), and results showed a significant reduction in the number of admissions (Fig. 4a). In within-group comparisons, the non-HFNT PR (2.59 ± 0.80 vs. 0.60 ± 0.82 times/year) and HFNT PR groups (2.27 ± 0.70 vs. 0.41 ± 0.67 times/year) had significantly decreased AE times at 1 year after the PR intervention, and no significant change was found in the between-group comparisons (Fig. 4b).

## Discussion

This study showed some significant outcomes after HFNT early PR in patients with AECOPD: improved exercise tolerance in the 6MWD after 4 and 12 weeks of the PR program, and decreased dyspnea sensation in mMRC and inflammation as evidenced by the CRP level after 12 weeks of PR. Moreover, a reduction in the length of hospitalization was achieved with PR at the 1-year follow-up; HFNT PR patients showed even better trends in QOL, reduced air trapping, and lower lung hyperinflation than non-HFNT PR patients, but the differences were not statistically significant between the two groups.

The first evidence of PR in acute respiratory patients was published by Trooster and colleagues: AECOPD patients who received training showed improvements in quadriceps force and 6MWD at discharge, and improvements were also documented at 1 month of follow-up [30]. The 6MWD is accepted as a good outcome measure after interventions such as PR [31, 32]. Our study had similar findings of HFNT PR patients exhibiting improved exercise tolerance as assessed by the 6MWD than non-HFNT PR patients after 4 and 12 weeks of PR. This opens up new possibilities for early rehabilitation treatment of patients hospitalized for AECOPD [33]. A systematic review suggested that PR after COPD exacerbation may reduce hospital admissions and mortality, and may improve the health-related QOL [34]. Our study reports decreased AE hospital admissions and an improved QOL including in the physical and functional domains from BODE within the two PR groups, although there were no significant differences in between-group comparisons.

The BODE index characterizes a multistage functional scoring system for COPD by means of a simple scale and requires no special equipment [35, 36]. Symptoms are the cornerstone for diagnosing AE of COPD. Dyspnea represents the most disabling symptom of COPD; the mMRC dyspnea scale is simple to administer and correlates with scores of health status [37]. In this study, after PR there were significantly decreased mMRC dyspnea levels at 4 and 12 weeks, and most patients improved above the MCID in the mMRC. Although HFNT PR significant ameliorated airflow obstruction in terms of FEV1 at 12 weeks, there were no significant differences in between-group comparisons, which is probably related to the small sample size and indicates the need for longer-term follow-up.

CAT impacts a patient’s QOL [38], is responsive to treatment [39], and provides relevant prognostic information [40]; therefore, its use is advocated for assessing PR during AECOPD. In our study, after PR, there were significant improvements in the QOL at 4 and 12 weeks, and most patients improved above the MCID in CAT, although there were no significant differences in between-group comparisons.

Lung hyperinflation limits the expiratory flow in patients with COPD and contributes to dyspnea and activity limitations [41]; it has become an important therapeutic target in symptomatic COPD patients [42]. In our study, respiratory therapists were taught to use the pulse-lip/diaphragm breathing technique, relaxation exercises, and combined HFNT during exercise. HFNT patients showed better trends of reduced air trapping in delta IC after exercise compared to non-HFNT PR patients.

HFNT provides warmed humidified air administered through slightly enlarged nasal prongs. HFNT reduces the minute volume, lowers the respiratory rate, and decreases the work of breathing [21, 43], and it leads to a flow-dependent reduction in PaCO_2_ [21]. When we analyzed data on arterial blood gases (pH, PaO_2_, PaCO_2_, and HCO^3−^), our results of reduced PaCO_2_ levels were similar within each group, but did not significantly differ between the two groups at the end of the PR program. It is well known that AECOPD may be triggered by infection with bacteria or viruses or by noninfectious environmental or internal factors. Patients with AECOPD also display heterogeneous inflammation, and inflammatory markers such as CRP [44] as a kind of quantitative indicator are widely used in judging AE and for assessing prognoses [45]. Serum CRP may provide prognostic information about morbidity and mortality in COPD patients because of relationships among CRP, interleukin (IL)-6, exercise tolerance, and the health status [46, 47]. Our study proved that an HFNT PR program reduced CRP levels better than a non-HFNT PR program, and utilizing HFNT during exercise in the COPD PR program might help avoid fatigue, decrease exercise limitations related to the excessive load placed on inspiratory muscles, and reduce systemic inflammation.

Our study had some limitations. First, there was a small simple size, and only one hospital participated in the study. Second, too few women were recruited, and recent data indicate that female COPD patients may have a higher number of hospitalizations with a more-prolonged length of stay [48]. Third, pharmacological treatment was not standardized, although there were no differences at the baseline assessment, it must be acknowledged that this might have influenced patients’ recovery. Fourth, correlations of laboratory blood samples were not assessed for COPD inflammatory cytokines and oxidative markers such as IL-8, TNF-α, 8-isoprostant, and so on.

## Conclusions

High-flow nasal therapy during exercise training in early pulmonary rehabilitation is feasible for hospitalized patients with severe AECOPD who are profoundly intolerant of exercise training. Exercise training with HFNT can support an increased exercise capacity, decreased dyspnea sensations, and reductions in systemic inflammatory biomarkers in hospitalized patients with severe AECOPD. Our study also raises the possibility that HFNT during exercise enhances the training effect through decreased lung hyperinflation and increased pulmonary function.

## Data Availability

All data generated or analyzed during this study are included in this published article.

## Abbreviations

6MWD: 6-min walking distance
ABG: Arterial blood gas
AE: Acute exacerbation
ATS: American Thoracic Society
BMI: Body-mass index
BODE index: Body-mass index, degree of airway obstruction and dyspnea, and exercise capacity
BP: Blood pressure
CAT: COPD assessment test
COPD: Chronic obstructive pulmonary disease
CRP: C-reactive protein
CVD: Cardiovascular disease
FEV1: Forced expiratory volume in the first second
FVC: Forced vital capacity
GERD: Gastroesophageal reflux disease
HFNT: High-flow nasal therapy
HR: Heart rate
IC: Inspiration capacity
IL-6: Interleukin-6
IL-8: Interleukin-8
MCID: Minimal clinically important difference
mMRC: Modified Medical Research Council
NIMV: Non-invasive mechanical ventilation
PaCO2: Partial pressure of carbon dioxide
PaO_2_: Partial pressure of oxygen
PR: Pulmonary rehabilitation
QOL: Quality of life
RR: Respiration rate
SD: Standard deviation
WBC: White blood cells
